# Supplementary figures and images for: The Long and Viscous Road: Uncovering Nuclear Diffusion Barriers in Closed Mitosis
Source: PLoS Comput Biol. 2014 Jul 17;10(7):e1003725. doi: 10.1371/journal.pcbi.1003725 (PMC4102450; doi:10.1371/journal.pcbi.1003725)

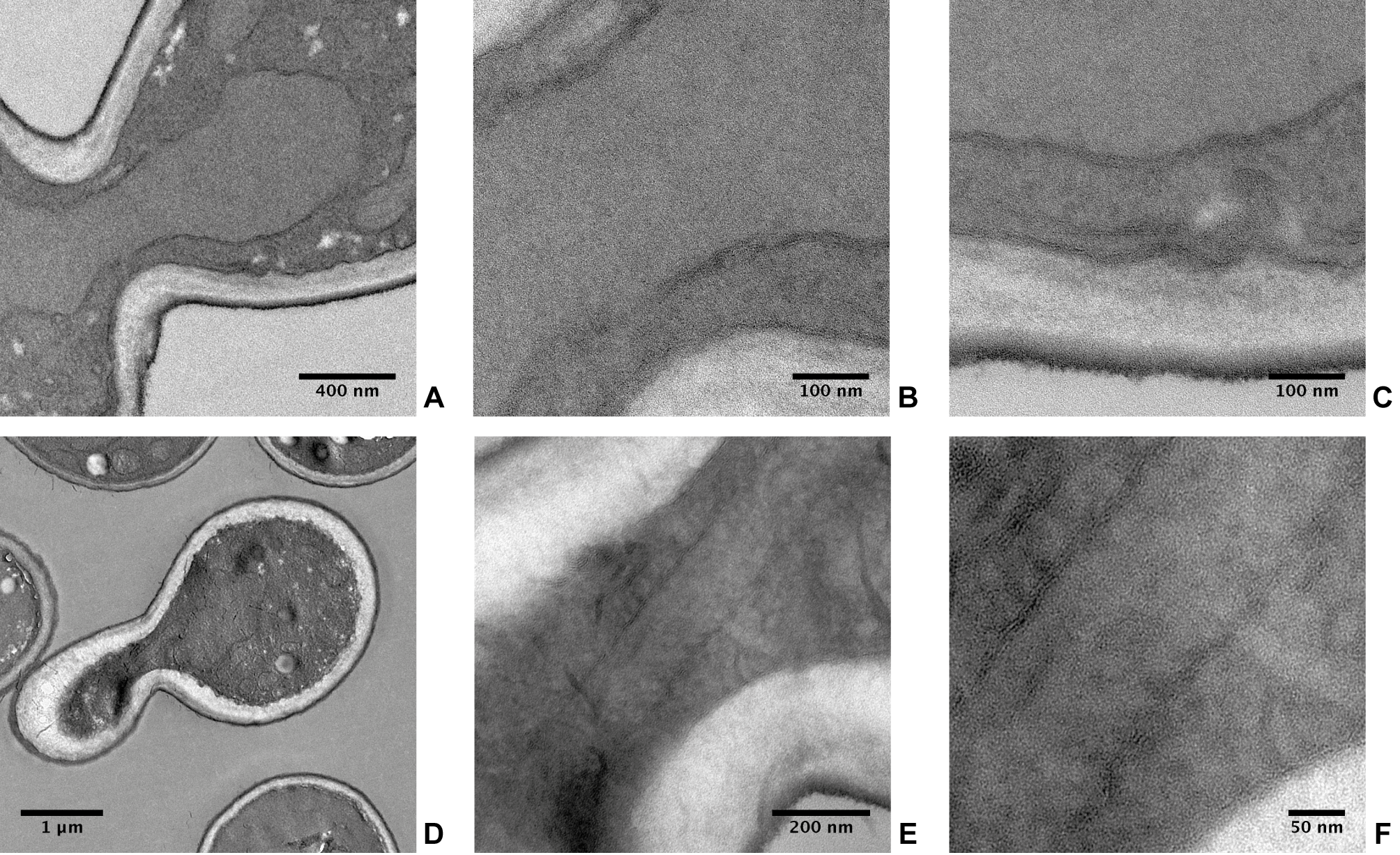

Supplement: Figure S3 — TEM images of yeast nuclei during anaphase. Each row, from top to bottom, shows cells in (A, B, C) early anaphase and (D, E, F) late anaphase. Zoomed areas from left column are shown in centre and right columns: early anaphase: (B) bud neck and (C) daughter nuclear lobe; late anaphase: (E) whole bridge and (F) bridge at neck. The average thickness of perinuclear space ( in Fig. 1, measured between the phospholipid heads of inner lipid leaflets facing the periplasm) was 22±6 nm at nuclear lobes (regardless of the mitotic stage) and 13±4 nm at the connecting bridge (in late anaphase). The staining protocol used was the same as in [65]. (TIF) [file pcbi.1003725.s003.tif]

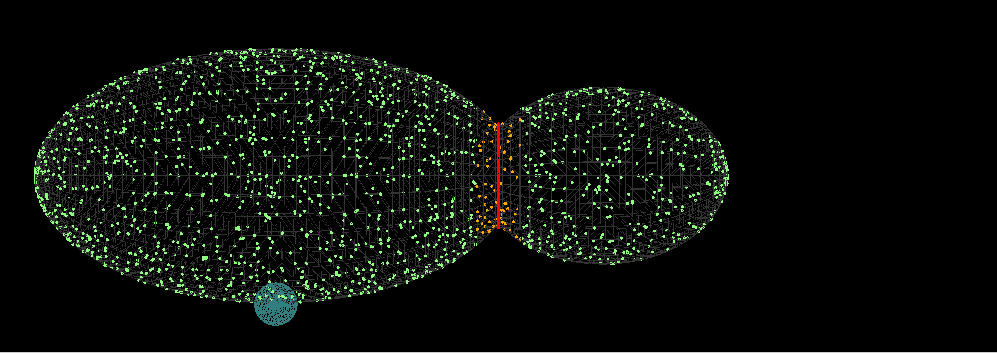

Supplement: Movie S1 — Simulation of GFP-Src1 diffusion in an EA nucleus. A protein ring (n = 200) and a specialised lipid ring domain at the neck (width = 300 nm) constrain lateral diffusion. The bleaching reaction begins at time t = 0 s, and occurs only at the bleach spot. The whole model is rotated 180° around the spindle axis and the mitotic axis to ease visualization of its 3D shape. Colour code: bleached (purple), non-bleached (green), bleached and non-bleached at domain (orange), proteins at ring (red). All molecular sizes have been doubled to ease visualization. (GIF) [file pcbi.1003725.s011.gif]

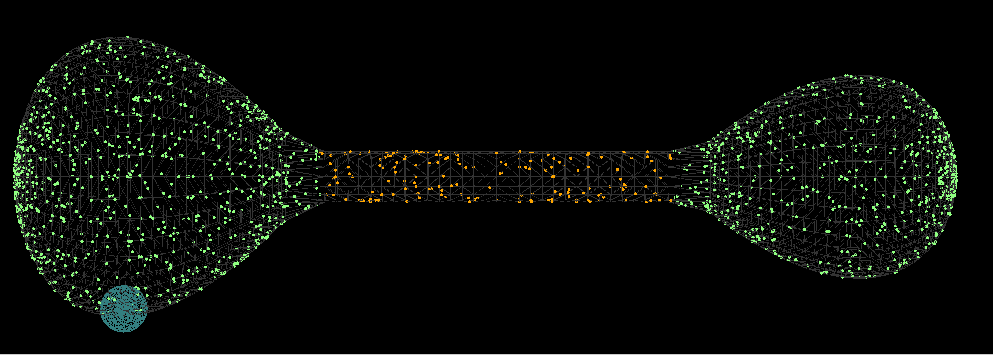

Supplement: Movie S2 — Simulation of Nsg1-GFP diffusion in an LA nucleus. A specialised lipid domain spanning the entire bridge length constrains lateral diffusion. The bleaching reaction begins at time t = 0 s, and occurs only at the bleach spot. The whole model is rotated 180° around the spindle axis and the mitotic axis to ease visualization of its 3D shape. Colour code: bleached (purple), non-bleached (green), bleached and non-bleached at domain (orange). All molecular sizes have been doubled to ease visualization. (GIF) [file pcbi.1003725.s012.gif]

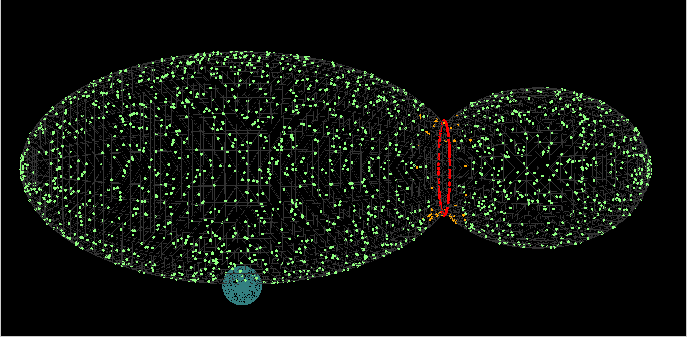

Supplement: Movie S3 — Simulation of GFP-Src1 FLIP experiments in an EA nucleus. A protein ring (n = 200) and a specialised lipid ring domain at the neck (width = 300 nm) constrain lateral diffusion. The whole model is slightly tilted to ease visualization of the protein ring. Colour code: bleached (dark green), non-bleached (light green), bleached and non-bleached at domain (orange), proteins at ring (red). All molecular sizes have been doubled to ease visualization. (GIF) [file pcbi.1003725.s013.gif]

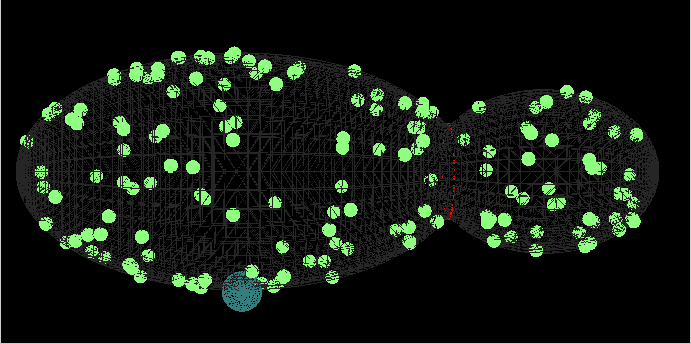

Supplement: Movie S4 — Simulation of NPC FLIP experiments in an EA nucleus. A protein ring (n = 200) and a specialised lipid ring domain at the neck (width = 300 nm) constrain lateral diffusion. The whole model is slightly tilted to ease visualization of the protein ring. Colour code: bleached (dark green), non-bleached (light green), bleached and non-bleached at domain (orange), proteins at ring (red). Both the ONM and INM are shown, with all NPCs diffusing within the perinuclear space between them. (GIF) [file pcbi.1003725.s014.gif]
