# Supplementary material for: The Long and Viscous Road: Uncovering Nuclear Diffusion Barriers in Closed Mitosis
Source: PLoS Comput Biol. 2014 Jul 17;10(7):e1003725. doi: 10.1371/journal.pcbi.1003725 (PMC4102450; doi:10.1371/journal.pcbi.1003725)
Supplement: Table S1 — Diffusing protein parameters for spatial-stochastic simulations. Protein numbers were taken from the Yeast GFP Fusion Localization Database [11], [52]. Source references for protein sizes are indicated. Sizes used in simulations take into account GFP fusion. An exception is the NPC, given that Nup49 is buried in its inner rings. (PDF) [file pcbi.1003725.s009.pdf]

Table S1. Diffusing protein parameters for spatial-stochastic simulations.

Molecular species	Initial number	Diffusion rate	Estimated size (including GFP)	Reference
GFP	-	-	(W x L) 2.4 x 4.2 nm	[1]
TetR-GFP	5000 @ nucleoplasm	1.9 $\mu m^2/s$	4 nm (8 nm)	[2]
NPC (Nup49-GFP)	150 @ periplasm	0.2 $\mu m^2/s$	98 nm	[3]
Nsg1-GFP	1900 @ ONM	0.3 $\mu m^2/s$	~ 4.2 nm (8 nm)	[4]
GFP-Src1	2100 @ INM	0.3 $\mu m^2/s$	~ 6 nm (10 nm)	[4]

References

1. Ormö M, Cubitt AB, Kallio K, Gross LA, Tsien RY, et al. (1996) Crystal structure of the Aequorea victoria green fluorescent protein. Science 273: 1392-1395.
2. Hinrichs W, Kisker C, Duvel M, Muller A, Tovar K, et al. (1994) Structure of the Tet repressor-tetracycline complex and regulation of antibiotic resistance. Science 264: 418-420.
3. Alber F, Dokudovskaya S, Veenhoff LM, Zhang W, Kipper J, et al. (2007) The molecular architecture of the nuclear pore complex. Nature 450: 695-701.
4. Erickson HP (2009) Size and shape of protein molecules at the nanometer level determined by sedimentation, gel filtration, and electron microscopy. Biol Proced Online 11: 32-51.
