# Supplementary material for: The Long and Viscous Road: Uncovering Nuclear Diffusion Barriers in Closed Mitosis
Source: PLoS Comput Biol. 2014 Jul 17;10(7):e1003725. doi: 10.1371/journal.pcbi.1003725 (PMC4102450; doi:10.1371/journal.pcbi.1003725)
Supplement: Table S2 — Estimated parameters of sphingolipid domains. Nuclear membranes estimated thickness, viscosity, viscous drag, diffusion coefficient and permeability of the sphingolipid domain. Values outside and inside of the sphingolipid domain are specified where applies. All values were estimated using the Petrov-Schwille model. (PDF) [file pcbi.1003725.s010.pdf]

Table S2. Estimated parameters of sphingolipid domains.

Parameter	Description	Value	Value outside of sphingolipid domain	Value inside of sphingolipid domain
h_{ONM}, h_{INM}	Membrane thickness	-	4 nm	5.4 nm
h_p	Perinuclear space thickness at connecting bridge	13 ± 4 nm	13 nm	If domain only at ONM: 12.3 nm<br>If domain at ONM and INM: 11.6 nm
μ_c, μ_n, μ_p	Bulk viscosities of cytoplasm, nucleoplasm and periplasm	11.967 x 10^{-4} Pa s	-	-
η_{ONM}	Surface viscosity of ONM	-	7.5333 x 10^{-9} Pa s m	2.6826 x 10^{-8} Pa s m
η_{INM}	Surface viscosity of INM	-	7.2424 x 10^{-9} Pa s m	2.5976 x 10^{-8} Pa s m
η_{NE}	Surface viscosity of NE $\eta_{ONM} + \eta_{INM} + \mu_p h_p$	-	1.4791 x 10^{-8} Pa s m	If domain only at ONM: 3.4083 x 10^{-8} Pa s m<br>If domain at ONM and INM: 5.2816 x 10^{-8} Pa s m
γ_{Nsg1}	Viscous drag experienced by Nsg1-GFP	-	1.3951 x 10^{-8} Pa s m	4.1853 x 10^{-8} Pa s m
γ_{Src1}	Viscous drag experienced by GFP-Src1	-	1.3951 x 10^{-8} Pa s m	4.1853 x 10^{-8} Pa s m
γ_{NPC}	Viscous drag experienced by NPC	-	2.0926 x 10^{-8} Pa s m	If domain only at ONM: 7.3943 x 10^{-8} Pa s m<br>If domain at ONM and INM: 1.0655 x 10^{-7} Pa s m
$D_{Nsg1\text{-}GFP}$	Effective diffusion rate of Nsg1-GFP	-	0.3 μm^2/s	0.1 μm^2/s
$D_{GFP\text{-}Src1}$	Effective diffusion rate of GFP-Src1	-	0.3 μm^2/s	0.1 μm^2/s
D_{NPC}	Effective diffusion rate of NPC	-	0.2 μm^2/s	Domain only at ONM: 0.0566 μm^2/s<br>Domain at ONM and INM: 0.0393 μm^2/s
